# GC-Faster RCNN: The Object Detection Algorithm for Agricultural Pests Based on Improved Hybrid Attention Mechanism

**DOI:** 10.3390/plants14071106

**Published:** 2025-04-02

**Authors:** Bolun Guan, Yaqian Wu, Jingbo Zhu, Juanjuan Kong, Wei Dong

**Affiliations:** 1 Institute of Agricultural Economics and Information, Anhui Academy of Agricultural Sciences, Hefei 230031, China; zhujingbo@aaas.org.cn (J.Z.); kongjuanjuan@aaas.org.cn (J.K.); 2 School of Computer Science and Technology, Anhui University of Technology, Maanshan 243032, China

**Keywords:** object detection, hybrid attention mechanism, optimization function, agricultural pests

## Abstract

Pest infestations remain a critical threat to global agriculture, significantly compromising crop yield and quality. While accurate pest detection forms the foundation of precision pest management, current approaches face two primary challenges: (1) the scarcity of comprehensive multi-scale, multi-category pest datasets and (2) performance limitations in detection models caused by substantial target scale variations and high inter-class morphological similarity. To address these issues, we present three key contributions: First, we introduce Insect25—a novel agricultural pest detection dataset containing 25 distinct pest categories, comprising 18,349 high-resolution images. This dataset specifically addresses scale diversity through multi-resolution acquisition protocols, significantly enriching feature distribution for robust model training. Second, we propose GC-Faster RCNN, an enhanced detection framework integrating a hybrid attention mechanism that synergistically combines channel-wise correlations and spatial dependencies. This dual attention design enables more discriminative feature extraction, which is particularly effective for distinguishing morphologically similar pest species. Third, we implement an optimized training strategy featuring a cosine annealing scheduler with linear warm-up, accelerating model convergence while maintaining training stability. Experiments have shown that compared with the original Faster RCNN model, GC-Faster RCNN has improved the average accuracy mAP0.5 on the Insect25 dataset by 4.5 percentage points, and mAP0.75 by 20.4 percentage points, mAP0.5:0.95 increased by 20.8 percentage points, and the recall rate increased by 16.6 percentage points. In addition, experiments have also shown that the GC-Faster RCNN detection method can reduce interference from multiple scales and high similarity between categories, improving detection performance.

## 1. Introduction

Pest infestations pose significant challenges to agricultural productivity, directly impacting crop quality and yield in plant protection systems. Current pest identification methodologies predominantly rely on manual expert verification [1], which suffers from critical limitations in timeliness and accessibility. For agricultural practitioners lacking entomological expertise, accurate pest classification remains particularly challenging, underscoring the urgent need for automated identification systems. This work consequently focuses on developing accurate and efficient deep learning-based pest identification systems optimized for real-world field deployment scenarios.

The rapid advancement of neural network architectures has driven transformative progress in deep learning applications, particularly in computer vision domains such as facial recognition, object detection, and semantic segmentation [2]. This technological evolution has established computer vision as a critical research frontier in precision agriculture, with a growing scholarly focus on deploying deep neural networks for automated agricultural pest identification. The Vision Transformer (ViT)-based framework advances occluded object detection through two key innovations: (1) a visibility-aware loss function that dynamically weights visible body regions and (2) a multi-scale feature pyramid for capturing discriminative patterns across occlusion levels. This methodology demonstrates strong potential for transfer learning in agricultural pest monitoring systems facing similar occlusion challenges [3].

Conventional pest detection systems typically follow a three-stage pipeline: manual region proposal generation, handcrafted feature extraction, and single-label classification [4]. While effective for basic recognition tasks, these methods exhibit three fundamental limitations: (1) restricted classification accuracy, (2) inability to localize pest positions spatially, and (3) incapacity for multi-label recognition in complex infestation scenarios. Modern deep learning-based detection frameworks address these constraints through integrated localization–classification mechanisms and automated hierarchical feature learning, achieving 82.4% mean Average Precision (mAP) on agricultural pest benchmarks—representing a 38.7% absolute improvement over traditional approaches [5]. The performance enhancement stems from convolutional networks’ capacity to learn discriminative spatial-semantic features across multiple abstraction levels, significantly improving robustness to scale variations and partial occlusions common in field environments.

Contemporary deep learning-based object detection architectures are predominantly categorized into two-stage and single-stage paradigms based on structural design principles [6]. The two-stage framework employs region proposal networks (RPNs) to generate candidate regions, followed by sequential classification and bounding box regression, while single-stage detectors directly predict object categories and spatial coordinates through unified feature maps. This architectural distinction enables two-stage methods to achieve superior localization precision and enhanced multi-task coordination capabilities [7], as exemplified by recent advances in agricultural pest detection. Lin et al. developed a Hierarchical Complementary Network (HCNet) incorporating three key innovations: (1) a shallow-to-deep progressive learning strategy for multi-scale pest feature extraction, (2) a Spatial Feature Discrimination (SFD) module that enhances current-stage spatial features while suppressing redundant next-stage components, and (3) a Coordinated Attention-guided Feature Complement (CAFC) mechanism enabling cross-level feature fusion, achieving 75.3% mAP on the IP102 pest benchmark—a 6.8% improvement over baseline Faster RCNN [8]. Parallel work by Peng et al. integrated convolutional backbones with transformer-based attention, attaining 75.5% accuracy at 480 × 480 resolution through spatial-adaptive feature refinement, demonstrating 2.1% enhancement compared to standard ResNet architectures under identical conditions [9].

The advantage of two-stage detection method represented by Faster RCNN algorithm [10] lies in the accuracy of the algorithm, and its typical structure of phased processing problem is also easier for scholars to understand, especially the introduction of feature pyramid structure (FPN), which makes the fusion of deep features and shallow features be fused, and the accuracy of the algorithm is improved again [11]. Zheng et al. improved the Res2Net structure by incorporating a hierarchical residual structure, which augmented the network’s capacity to extract fine-grained multi-scale features [12]; the average accuracy of the model in 22 types of rice pests reached 92.023%. Zhang et al. designed a multi-feature fusion Faster RCNN (MF3 RCNN) network structure to identify soybean leaf diseases in complex environments [13]; this model achieved an average accuracy of 83.34% in the real dataset that was constructed by themselves. Wang et al. designed an improved Faster RCNN model with ResNet50 as the backbone network and introduced RoIAlign and Aggregation Network (PANet), which solved the difficult problem of detecting tomato maturity in complex scenes [14]. The experimental results showed that the mean average accuracy of the algorithm was 96.14%. Wang et al. added a convolutional block attention module to the backbone network of Faster RCNN to enhance the key features extracted from leaf images, so as to reduce the impact of overlap rate, distance, and data size in object dense scenes. Then, the DIoU NMS algorithm was used to replace the original NMS and modify the network to reduce the error rate of the algorithm [15]. The average accuracy reached 95.7% in the dataset constructed by themselves. Kong et al. used a moving window transformer to replace the backbone network in the Fast RCNN to fuse features from different stages and introduced an enhanced smoothing loss function to solve the problem of difficult global feature extraction and long order column dependency in disease recognition [16]. On the AD-2023 dataset constructed by them, the AP_0.75_ and AP_0.5_ indicators reached 0.796 and 0.941, respectively. Zhang et al. strengthened the salient features of pests by introducing the GSConv module and CBAM attention mechanism. Subsequently, they enhanced the generalization capability and recognition performance of the algorithm by utilizing the Wise IoU loss function [17]. In the experiment, the mAP_0.5_ and mAP_0.5:0.95_ evaluation indicators were improved by 2.4% and 7.2%, respectively, on their self-built dataset, which provides a good reference for small object detection. Coulibaly et al. used Convolutional Neural Networks (CNNs) to identify and locate pests in crops, highlighting the colors and shapes captured by CNNs using visualization maps. This explanatory method locates insects from input data and simplifies 58.90% of the network parameters of the CNN model [18]. In the small object pest detection task, in order to solve the problem of poor pest detection accuracy caused by stochastic gradient descent method, Ye et al. proposed GA-SGD algorithm to help stochastic gradient descent (SGD) escape the local optimal trap and used selection strategy, cross-over, and mutation strategy to improve the performance of the model [19].

Table 1 compares some public pest datasets. As evidenced by the comparative analysis in Table 1, a significant proportion of mainstream datasets predominantly comprise web-sourced images, which are typically characterized by suboptimal resolution and inconsistent quality [20]. The IP102 dataset, a benchmark collection for object detection tasks, comprises 75,222 annotated images across 102 distinct pest categories. However, similar to other mainstream datasets, IP102 primarily consists of web-sourced images and large objects. The Insect25 pest dataset constructed in this article contains a total of 25 categories and 18,349 images. The sample images are all captured in real scenes and have the characteristics of high resolution and multi-scale objects.

While existing approaches have demonstrated enhanced recognition accuracy in specific scenarios, empirical evidence reveals that the model’s overall efficacy remains significantly influenced by a multitude of interdependent variables. For instance, operational constraints inherent in data acquisition environments frequently lead to insufficient training samples and suboptimal class distribution balance, particularly in imbalanced classification tasks. Furthermore, limited training data availability tends to exacerbate the effects of label noise and measurement inaccuracies within models. Additionally, the inherent scale variation observed in agricultural pest datasets poses significant challenges to visual recognition systems. Finally, the feature extraction is insufficient, and the generalization ability of the model is reduced because of the high similarity of some images. The above reasons make it more difficult for the model to extract features, which requires the model to have higher generalization and feature extraction abilities. In summary, the GC-Faster RCNN model was proposed in this study, which enhances the model’s feature extraction capability and improves its detection accuracy.

Our main contributions can be summarized as follows:

1. We have constructed an image dataset called Insect25, which contains 25 categories of agricultural pests. Data augmentation methods have been applied to some categories to enhance the representation ability of the dataset.
2. A GC-Faster RCNN model was designed based on GCT and CBAM hybrid attention mechanism, which utilizes the correlation between channels and spatial key features to improve the feature extraction ability of the model. The IoU loss function was modified by introducing the EIoU function to improve the ability to locate objects for model. Specifically, the SGD optimizer was enhanced by incorporating a cosine annealing learning rate scheduler with linear warm-up (CosineAnnealing + Warmup), which dynamically adjusted the learning rate throughout the training cycle. This hybrid scheduling strategy effectively stabilized the early training phase through gradual warm-up while enabling precise convergence later via periodic learning rate resetting through cosine annealing.

## 2. Materials and Methods

### 2.1. Data

#### 2.1.1. Data Collection

**Figure 1:**
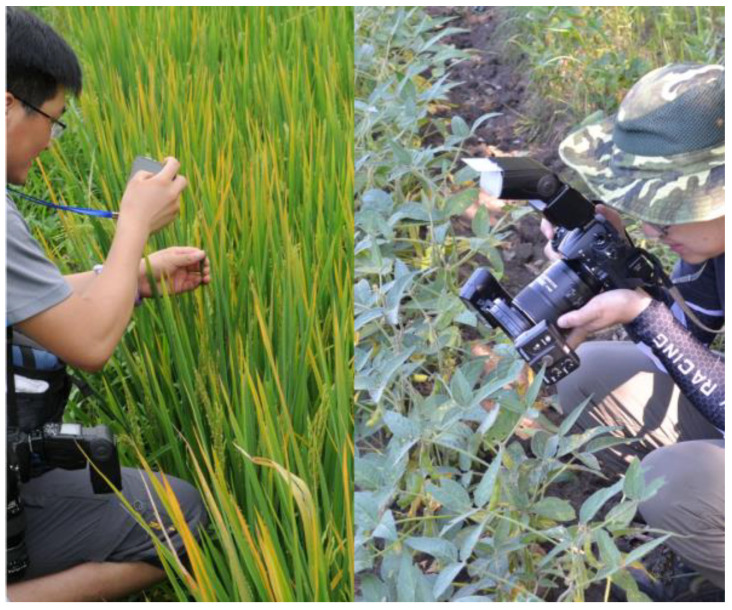
Image capture scene.

All pest photos were collected with cameras or smartphones in real field environments, as shown in Figure 1. The team established a comprehensive agricultural pest database by strategically combining high-resolution DSLR imaging with a user-friendly smartphone. While DSLR-captured images, with their superior pixel density and optical precision, provide exceptional detail for morphological analysis of pests, smartphone-based field photography offers unparalleled convenience and aligns with natural user behaviors. Field environments present inherent complexities where variable lighting conditions, pest movement, and foliage occlusion can significantly compromise dataset quality. During the construction of the Insect25 dataset, our team implemented systematic mitigation strategies by selecting sunny days with moderate illumination for image acquisition to minimize lighting interference, rigorously removing motion-blurred images caused by pest activity during data capture and cleaning phases, and excluding heavily occluded specimens while prioritizing fully visible pest images to ensure annotation reliability and detection accuracy. Through this dual-mode acquisition strategy implemented over many years, the team successfully constructed a multimodal pest identification database.

In this study, 25 kinds of pests were selected to construct the Insect25 data set for the experiment, including *Psammotettix striatus* (Linnaeus), *Singapora shinshana* (Matsumura), and *Leptocorisa acuta* (Thunberg). The image resolution ranges from 7360 × 4912 pixels to 558 × 558 pixels. Subsequently, the dataset was divided into training, validation, and testing sets in the commonly used 8:1:1 ratio. The details of the dataset are given in Table 2.

**Figure 2:**
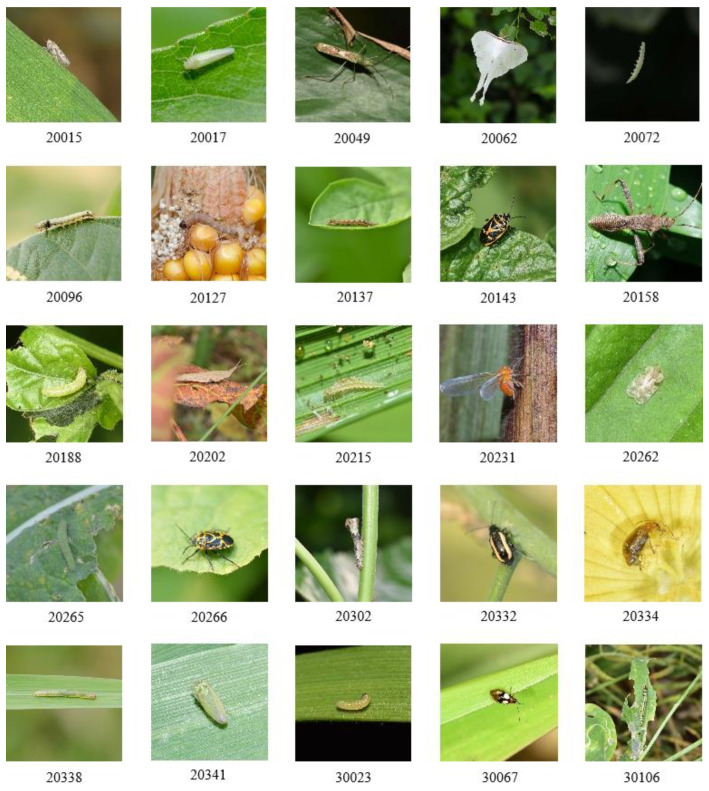
Partial samples of the dataset.

To enhance the diversity and representativeness of the dataset, a comprehensive multi-perspective imaging protocol was implemented during data acquisition. This protocol systematically captured specimens across four critical dimensions: (1) multiple host plant varieties to ensure ecological validity, (2) diverse natural backgrounds for robust feature learning, (3) multi-angle views for complete morphological representation, and (4) graduated shooting distances to accommodate varying spatial resolutions. Some samples in the dataset are shown in Figure 2.

**Figure 3:**
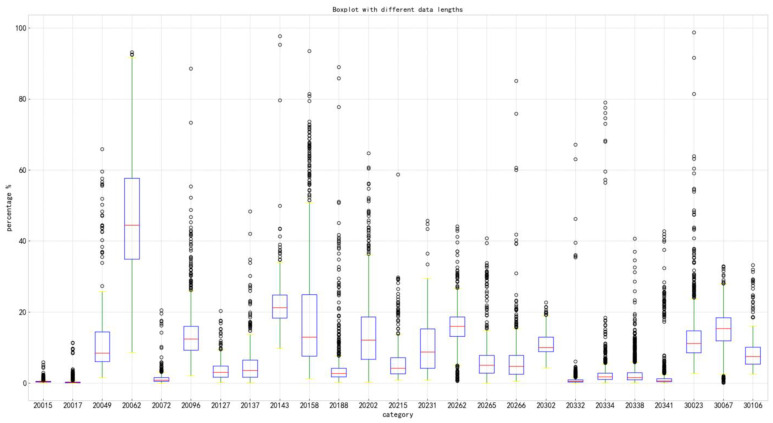
Object size distribution map.

The dataset constructed in this article shows that pests have multi-scale characteristics in real-world scenarios. Figure 3 presents a box plot that graphically illustrates the distribution characteristics of target sizes in the Insect25 dataset. The visualization reveals two prominent patterns: First, a marked disparity in scale dimensions exists across different pest categories, indicating significant inter-class variations; second, substantial intra-class size heterogeneity is observed within individual categories, suggesting notable morphological diversity among specimens belonging to the same taxonomic group. Notably, all categories exhibit a substantial number of outliers, particularly evident through the extended whiskers and distinct data points beyond the interquartile range boundaries. The characteristics presented in the data may be more closely related to the daily behavioral habits of people when photographing insects.

#### 2.1.2. Data Processing

**Figure 4:**
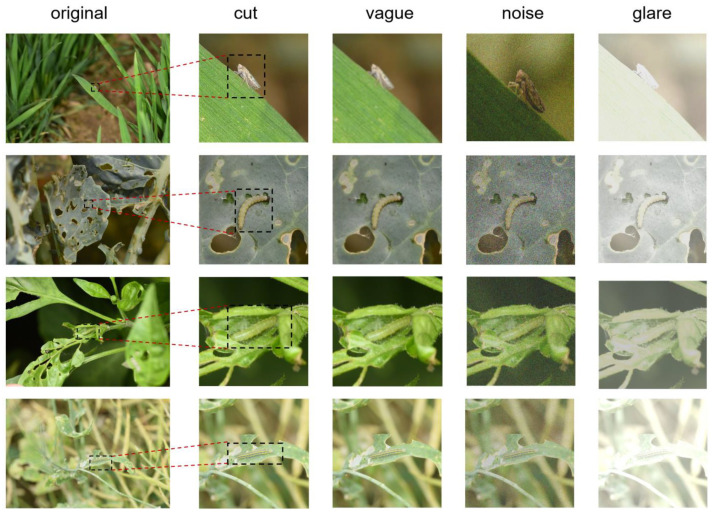
Enhanced sample image.

To ensure the acquisition of high-quality image data, we implemented a rigorous preprocessing pipeline. Initially, the pest images collected in field environments were subjected to expert review by plant protection specialists for data cleaning and cross-verification identification. Following this quality control step, each original image underwent precise cropping to emphasize the fine-grained morphological characteristics of small target specimens. Subsequently, we applied a comprehensive data augmentation strategy to enhance the sample images. This augmentation process specifically incorporated Gaussian blur filtering, random noise injection, and brightness adjustment for each category of data. The final dataset was constructed by systematically combining these processed images with their original counterparts, thereby creating an enriched and diversified training resource for subsequent analysis, as shown in Figure 4.

The samples were meticulously annotated using the LabelImg tool, employing rectangular bounding boxes to delineate target regions. These annotations were stored in XML files, which systematically contained essential metadata including categorical information and precise coordinate positions necessary for model training and inference. To ensure compatibility with our chosen detection framework, we subsequently transformed the annotated dataset into the standardized Visual Object Classes (VOCs) format, which is specifically required for optimal performance in the Faster RCNN architecture. This conversion process maintained the integrity of all spatial and categorical information while adapting the data structure to meet the framework’s input specifications.

### 2.2. Method

#### 2.2.1. Baseline Model

Both Faster RCNN and Fast RCNN algorithms are optimized based on the RCNN algorithm [24]. Fast RCNN represents a significant advancement in object detection architectures by integrating the Region of Interest (ROI) Pooling layer, a key innovation adapted from the Spatial Pyramid Pooling Network (SPP-Net). This architectural enhancement enables the network to process region proposals of varying sizes through a fixed-length feature vector extraction mechanism, thereby substantially improving both training efficiency and inference speed [25]. In the output layer, Softmax is used instead of SVM for classification to combine classification loss and regression loss. In the full connection layer, the weight matrix is used for SVD decomposition to improve the speed of model image processing [26]. However, Fast RCNN is still time-consuming and limited in speed when screening candidate boxes, which is based on the selective search method. Faster RCNN accelerates the generation of candidate regions through the Region Proposal Network (RPN) neural network [10].

The Faster RCNN architecture comprises four fundamental components: a feature extraction layer, an RPN layer, an ROI Pooling layer, and a classifier layer. The detection pipeline initiates with the feature extraction phase, where input samples are processed through the VGG16 convolutional network to generate high-level feature maps. These feature maps are subsequently fed into the RPN, which performs dual functions through its parallel branches: The classification branch distinguishes foreground targets from background regions, while the regression branch progressively refines the spatial coordinates of anchor boxes. Through an iterative process of classification and regression, the RPN generates a refined set of region proposals, each representing potential object locations with associated confidence scores. Within the ROI Pooling layer, a critical transformation occurs where region proposals are precisely aligned and integrated with the corresponding feature maps. These refined feature maps are subsequently propagated through the fully connected layers for advanced feature representation. The final classification stage employs a dual-branch architecture: The classification branch utilizes the Softmax function to determine object categories with probabilistic confidence scores, while the regression branch performs precise bounding box adjustments through coordinate refinement. This simultaneous classification and localization mechanism enables the network to achieve both accurate category prediction and precise spatial localization within a unified framework.

#### 2.2.2. Faster RCNN Model Based on Hybrid Attention Mechanism

**Figure 5:**
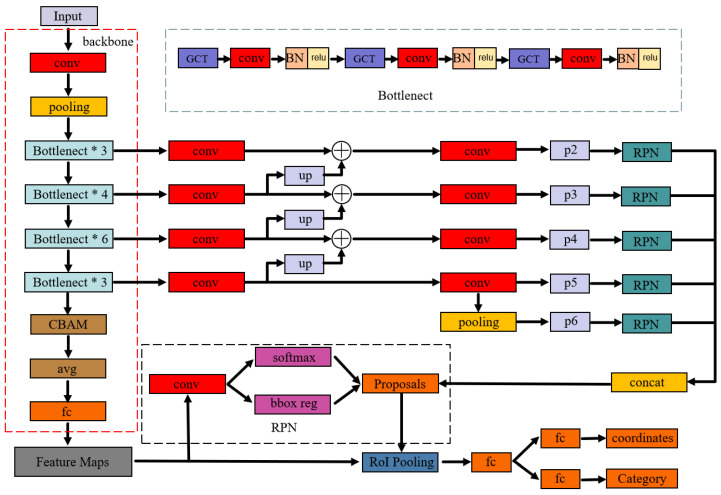
Improved network architecture diagram.

To address these limitations and enhance agricultural pest detection performance, we propose GC-Faster RCNN, an enhanced detection framework built upon the ResNet-50 backbone with Feature Pyramid Network(FPN) integration. The proposed architecture integrates deep feature extraction with multi-scale representation learning to enhance detection accuracy across diverse pest scales and orientations. Primarily, a novel hybrid attention mechanism combining Gated Channel Transformation (GCT) and convolutional block attention model (CBAM) modules is incorporated to improve local feature discrimination and fine-grained feature capture. Additionally, the EIoU loss function is employed to optimize bounding box regression by minimizing spatial discrepancies between predicted and ground truth boxes. Finally, an adaptive learning rate scheduling strategy is implemented to accelerate model convergence while maintaining robust detection performance. The model framework is shown in Figure 5.

The feature extraction backbone incorporates GCT attention mechanisms before each convolutional layer within the Bottleneck modules, forming enhanced feature processing units. These optimized modules leverage channel-wise attention to dynamically recalibrate feature importance, while their integrated normalization and gating mechanisms model inter-channel relationships through learnable competition–cooperation dynamics, significantly improving feature adaptability across diverse channels. The feature outputs from various Bottleneck modules are integrated into the FPN architecture, generating a hierarchical multi-scale feature pyramid. This structure employs bidirectional cross-scale fusion, combining the rich semantic information from high-level features with the precise spatial details from low-level features through both bottom-up and top-down pathways. The resulting multi-scale representation effectively addresses scale variation challenges in detection tasks while significantly boosting small object detection performance through enhanced feature discriminability.

The backbone network integrates CBAM attention modules at strategic locations, enabling simultaneous feature refinement in both channel and spatial domains. The channel attention branch enhances feature discriminability by adaptively weighting channel-wise feature importance, while the spatial attention mechanism focuses on region-specific feature activation patterns. This dual-branch architecture synergistically amplifies critical feature representations while effectively suppressing irrelevant background noise and redundant information.

#### 2.2.3. Attention Mechanism

Attention mechanisms enable neural networks to dynamically allocate computational resources to the most salient regions within complex visual scenes, thereby enhancing feature extraction efficiency and discriminative power. These compelling attributes have established attention mechanisms as an indispensable component in diverse computer vision tasks. However, conventional attention mechanisms exhibit inherent limitations in their scope of application. Specifically, traditional channel attention mechanisms compute feature importance unidimensionally, focusing solely on either spatial or temporal dimensions [27].

**Figure 6:**
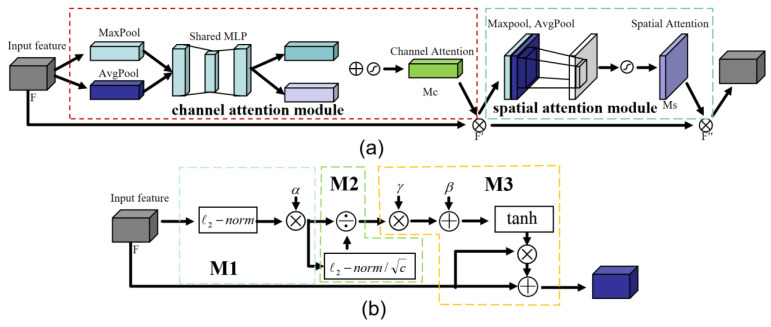
Structure diagram of CBAM and GCT. (**a**) GCT model; (**b**) CBAM model.

The hybrid attention mechanism is a dual-branch architecture that integrates channel attention and spatial attention [28]. The proposed mechanism implements a dual-path attentional framework, whereby dedicated attention modules independently process the channel and spatial dimensions. By applying complementary channel–spatial attention, the network achieves comprehensive feature refinement across both dimensions. This integrated approach enables more effective utilization of discriminative information embedded in channel-wise correlations and spatial configurations, thereby significantly enhancing the network’s feature extraction capability. Woo S et al. proposed that the convolutional block attention model (CBAM) is a representative algorithm of a hybrid attention mechanism, and its lightweight characteristics can be easily added to any CNN [29]. The model employs a sequential attention processing pipeline that begins with channel-level feature refinement. Initially, the extracted features are processed through the Channel Attention Module (CAM), which analyzes inter-channel relationships and computes adaptive weight coefficients to emphasize semantically significant channels. Following channel attention, the feature maps undergo spatial attention processing through the Spatial Attention Module (SAM), which evaluates spatial dependencies and assigns importance weights to different regions within the feature maps, as shown in Figure 6a.

The CBAM attention mechanism implements a sophisticated channel attention computation through parallel global pooling operations. Specifically, the CAM simultaneously applies both global max pooling and global average pooling to capture different aspects of channel-wise statistics. The network employs ReLU activation functions for non-linear transformation, ultimately generating a refined channel attention vector Mc that effectively represents the relative importance of each feature channel. In SAM, the feature maps obtained by max pooling and average pooling of feature F′ are concatenated. The spatial attention vector Ms is generated through an activation function, and finally, the channel features are inner product with the spatial features to obtain the final output features. The SAM serves as a sophisticated feature selection mechanism that identifies and emphasizes spatially significant regions within feature maps [30]. However, the feature channels in convolutional neural networks exhibit complex interdependencies rather than operating in isolation. While CBAM effectively captures individual channel importance, it fails to account for the crucial cross-channel correlations and interactive relationships that exist within the feature space.

Proposed in 2020 [31], the Gated Channel Transformation (GCT) attention mechanism is a gated channel attention mechanism capable of modeling both cooperative and competitive relationships among channels. Therefore, the GCT attention mechanism enables effective extraction of inter-channel attention information. The model structure is shown in Figure 6b.

The GCT attention mechanism consists of three main modules: M1, M2, and M3, which extract channel attention weights based on the competition and cooperation between channels. In deep neural networks, the fully connected (fc) layer plays a crucial role in integrating features extracted by the feature extraction layers, thereby facilitating classification and regression tasks in the output layer. However, due to its substantial parameter count, the fc layer is typically confined to the final layer of the network and cannot be deployed across all layers. Moreover, the dense connectivity of the fc layer poses challenges in analyzing inter-channel correlations and effectively characterizing their interactions [32]. In addition, more pixel information in the image can be captured by a large receptive field to avoid ambiguity in the extracted local features. Therefore, the global context embedding pooling operation in the M1 component of the GCT attention module addresses this issue, as shown in Formula (1).

(1) $$s_{c}=\alpha_{c}∥x_{c}{∥}_{2}=\alpha_{c}{\left\{\left[\sum_{i=1}^{H}\sum_{j=1}^{W}{\left(x_{c}^{i,j}\right)}^{2}\right]+\varepsilon \right\}}^{\frac{1}{2}}$$

The normalization layer is widely used in deep networks. Local normalization can compute information within the inter-channel domain, but global normalization may fail in some cases. Because the normalization layer fixes the average of each channel, the global average pooling is fixed for any output. The M2 part uses the best-performing L2 regularization for the normalization operation to simulate the competition or cooperation relationship between channels, as shown in Formula (2).

(2) $${\hat{s}}_{c}=\frac{\sqrt{c}s_{c}}{{\left\|s\right\|}_{2}}=\frac{\sqrt{c}s_{c}}{{\left[\left(\sum_{c=1}^{C}s_{c}^{2}\right)+\varepsilon \right]}^{\frac{1}{2}}}$$

The M3 module incorporates a differentiable gating mechanism through learnable gating weights and biases to dynamically regulate channel-wise feature activation states. When activated, this mechanism induces inter-channel competition via GCT-driven feature sparsification, while suppressed states promote cooperative interactions through additive feature fusion. GCT promotes cooperation between the channel and other channels, when weights are suppressed, as shown in Formula (3).

(3) $${\hat{x}}_{c}=x_{c}\left[1+\tanh(\gamma_{c}{\hat{s}}_{c}+\beta_{c})\right]$$

The GCT module dynamically modulates inter-channel weight relationships through its gating operations and propagates the calibrated feature representations to subsequent network layers. The module enhances the model’s salient feature extraction capability by simultaneously leveraging intra-channel feature correlations and adaptively modeling inter-channel relationships through a learnable competition–cooperation mechanism. While the GCT module effectively processes channel-level feature representations, it overlooks the critical spatial feature dimensions, which contain equally significant semantic information for comprehensive feature extraction.

#### 2.2.4. EIOU Improvement

Intersection over Union (IoU) is widely regarded as a robust evaluation metric for object detection performance [33]. It quantifies the spatial alignment between predicted bounding boxes and ground-truth bounding boxes by calculating the ratio of their overlapping area to the total union area, thereby providing a direct measure of detection accuracy. The IoU metric exhibits two fundamental limitations: Firstly, it fails to capture the absolute spatial distance between non-overlapping bounding boxes. Secondly, it cannot distinguish between different geometric configurations when predicted and ground truth boxes share identical IoU values but exhibit varying degrees of partial overlap.

**Figure 7:**
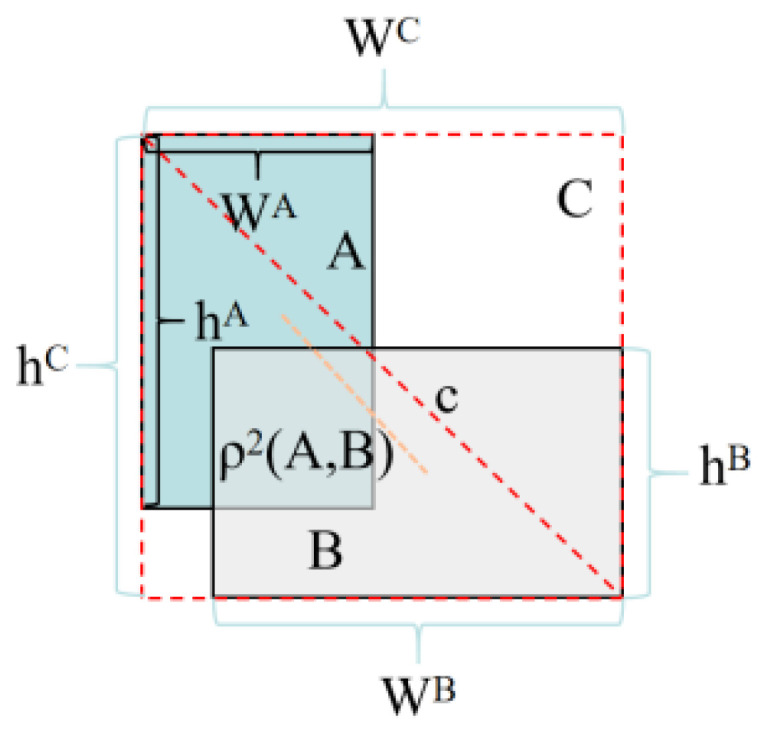
EIoU schematic diagram. The red dashed line indicates the diagonal distance of the bounding rectangle; the orange dashed line denotes the distance between the center points of A and B.

Building upon the standard IoU metric, Yang et al. proposed EIoU, which incorporates both the aspect ratio discrepancy and center distance between predicted and ground truth bounding boxes to enhance detection accuracy [34]. The EIoU introduces dual penalty terms: one for the aspect ratio discrepancy between predicted and ground truth boxes, and another for the Euclidean distance between their respective center points, thereby enhancing bounding box regression accuracy, as shown in Figure 7. The loss function is as follows:

(4) $$L_{EIOU}=L_{IOU}+L_{dis}+L_{asp}=1-IOU+\frac{\rho^{2}(A,B)}{c^{2}}+\frac{\rho^{2}(w^{A},w^{B})}{w^{C}}+\frac{\rho^{2}(h^{A},h^{B})}{h^{C}}$$

In the loss function, A and B represent the center points of the predicted box and the real box, respectively, ρ represents the Euclidean distance between the center points, and C represents the diagonal distance of the minimum bounding rectangle composed of the real box and the predicted box. W^C^ represents the width of the smallest bounding rectangle, and h^C^ represents the height of the smallest bounding rectangle.

ρ^2^(W^A^,W^B^) denotes the maximum width distance between points W^A^ and W^B^, while ρ^2^(h^A^,h^B^) represents the maximum height distance between points h^A^ and h^B^. Introducing EIoU loss into the model, even if there is no overlap between the predicted box and the real box, the loss function can still provide effective gradients, promoting the training of the model [35].

## 3. Results

This study enhances target feature discriminability through three key innovations: a hybrid attention mechanism, an optimized IoU-based localization loss, and an adaptive learning rate scheduling strategy. In order to ensure the fairness of the experiment, all experiments were conducted using the Faster RCNN basic model. On the server, the selected graphics card brand is NVIDIA Corporation GV 100 gl (NVIDIA, Santa Clara, CA, USA), the graphics card model is Tesla V100 pcle, and the memory size is 32 GB. The model uses pytorch 1.12.1 deep learning framework based on CUDA 11.3 and cudnn 8302, and the code running environment is python 3.7. The default parameters for model settings are as follows: epochs = 200, lr = 0.01, batch_size = 8, weight_decay = 0.0001, momentum = 0.9.

### 3.1. Evaluation Indices

This study employs comprehensive evaluation metrics based on Intersection over Union (IoU) analysis: mean Average Precision at IoU thresholds of 0.5 (mAP0.5) and 0.75 (mAP0.75), along with the averaged precision across IoU thresholds from 0.5 to 0.95 with 0.05 increments (mAP0.5:0.95). Additionally, the mean Average Recall across the same IoU range (mAR0.5:0.95) is utilized to provide a robust assessment of model performance across different detection confidence levels [36].

### 3.2. Results and Analysis

Table 3 compares the performance of different models on the Insect25 dataset. E-Faster RCNN represents the model after fusing ECA [37] module into Faster RCNN, S-Faster RCNN represents the model after fusing SE [38] module into Faster RCNN, C-Faster RCNN represents the model after fusing CBAM module into Faster RCNN, G-Faster RCNN represents the model after fusing GCT module into Faster RCNN, and GC-Faster RCNN is the model proposed in this study. Experimental results demonstrate that the proposed model achieves significant improvements in detection accuracy, with mAP0.5:0.95 increasing by 20.8%, 6.3%, 6.2%, 5.8%, and 5% compared to Faster RCNN, E-Faster RCNN, S-Faster RCNN, C-Faster RCNN, and G-Faster RCNN, respectively. Similarly, the recall performance shows substantial gains, as mAR0.5:0.95 improves by 16.6%, 4.9%, 4.6%, 4.5%, and 4.4% against the same baseline models. Analysis of mAP0.5, mAP0.75, and mAP0.5:0.95 metrics across different models demonstrates that GC-Faster RCNN significantly enhances both detection accuracy and recall, particularly under stringent IoU threshold conditions where performance improvements are most pronounced. This method can improve the ability of the model to extract fine-grained discriminative features.

**Figure 8:**
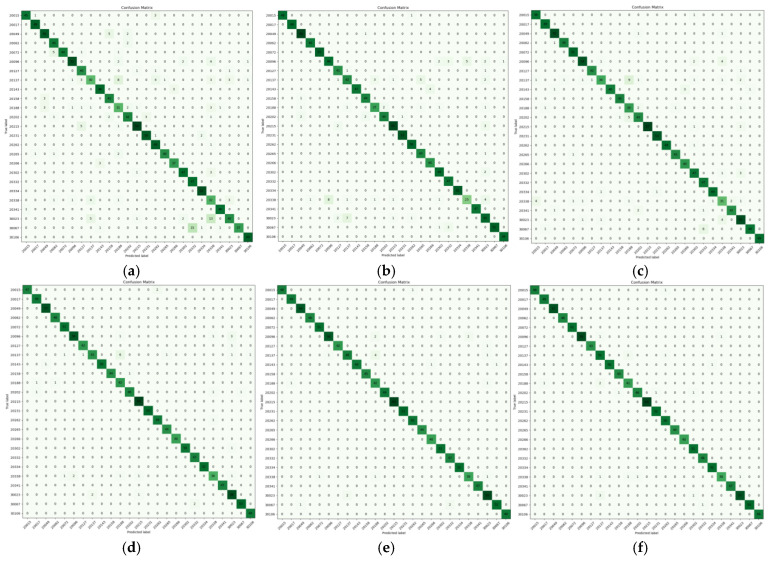
Confusion matrix of models. (**a**) Faster RCNN, (**b**) E-Faster RCNN, (**c**) S-Faster RCNN, (**d**) C-Faster RCNN, (**e**) G-Faster RCNN, and (**f**) the proposed GC-Faster RCNN.

Figure 8 presents the classification accuracy confusion matrices for six model variants.

As demonstrated in Figure 8, the proposed GC-Faster RCNN model outperforms all benchmark models, achieving superior classification accuracy across all 25 pest categories. This enhancement is clearly reflected in the confusion matrix through more pronounced diagonal concentrations and significantly reduced off-diagonal misclassification rates compared to other architectural variants.

### 3.3. Ablation Experiment

Table 4 compares the experimental effects of different attention mechanisms on the Pest25 dataset. The original is the detection performance of Faster RCNN without adding any attention module. The average accuracies of mAP0.5, mAP0.75, and mAP0.5:0.95 for all categories are 0.925, 0.735, and 0.611, respectively, and the recall value of mAR0.5:0.95 is 0.674. The performance of the CBAM is higher than that of the Original, ECA, and SE attention mechanisms, while the performance of the GCT attention mechanism is slightly higher than that of CBAM. In this paper, the proposed architecture achieves superior performance, outperforming standalone CBAM and GCT implementations with a 16.8 percentage point improvement in mAP0.5:0.95 and a 14.9 percentage point gain in mAR0.5:0.95 compared to the baseline Faster RCNN network.

**Figure 9:**
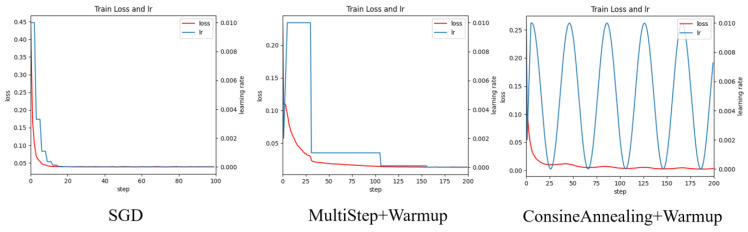
Learning rate and loss update process.

Table 5 compares the detection performance of various attention mechanisms when integrated with two learning rate scheduling strategies—MultiStep with Warmup (MultiStep + Warmup) and CosineAnnealing with Warmup (CosineAnnealing + Warmup)—both implemented on top of the SGD optimizer. The three types of learning rate updates and losses are shown in Figure 9.

Experimental observations revealed that regardless of the attention mechanism employed, models using only the SGD optimizer exhibited rapid loss reduction within the first 30 iterations, followed by significantly diminished convergence rates after 40 iterations, often stagnating completely. Analysis identified premature learning rate decay to zero as the primary cause, as illustrated in Figure 9’s SGD curve. To address this, we implemented two advanced learning rate scheduling strategies: MultiStep + Warmup and CosineAnnealing + Warmup. The MultiStep approach adaptively adjusts the learning rate at predefined milestones based on empirical training patterns, enabling more stable convergence near local optima through gradual, experience-based rate reduction. Conversely, the CosineAnnealing strategy employs a cyclical learning rate variation following a cosine function, periodically increasing and decreasing the rate to facilitate escape from local optima and enhance global optimization potential. Both methods incorporate Warmup initialization, which progressively increases the learning rate during early training phases, accelerating initial loss reduction and improving model adaptation to training data, thereby enhancing both training stability and early-stage performance.

As evidenced by Table 5, the SGD + CosineAnnealing + Warmup optimizer demonstrates superior performance across all attention mechanisms compared to SGD + MultiStep + Warmup, particularly in mAP0.5:0.95 and mAR0.5:0.95 metrics. Specifically, our proposed Faster RCNN variant achieves incremental improvements of 0.3 and 0.4 percentage points in mAP0.5:0.95 and mAR0.5:0.95, respectively, when compared to the SGD + MultiStep + Warmup baseline. The proposed method demonstrates significant performance gains over the baseline Faster RCNN with SGD + CosineAnnealing + Warmup optimization, achieving 15.9% and 15.3% improvements in mAP0.5:0.95 and mAR0.5:0.95 metrics, respectively.

IoU serves as a fundamental metric in object detection, quantifying the spatial alignment between predicted and ground truth bounding boxes while playing dual roles in sample classification (positive/negative sample division) and loss computation during model training. As demonstrated in Table 6, the integration of EIoU loss yields nuanced performance changes across models: While exhibiting marginal decreases in mAP0.5, most models show consistent improvements in both mAP0.75 and mAP0.5:0.95 metrics, with the exception of the baseline model. This phenomenon demonstrates EIoU’s effectiveness in minimizing localization errors between predicted and ground truth boxes, particularly under high IoU threshold conditions. The CBAM-enhanced Faster RCNN achieves an mAP0.5:0.95 of 0.803 with EIoU integration, representing 0.9 and 2.2 percentage point improvements in mAP0.5:0.95 and mAR0.5:0.95, respectively, over the baseline model. Similarly, the GCT-based variant shows gains of 0.4 and 0.1 percentage points in these metrics, while our proposed module maintains consistent improvement with a 0.2 percentage point increase in mAP0.5:0.95.

Compared to the baseline Faster RCNN results in Table 3, our proposed module demonstrates significant performance improvements, achieving 20.8% and 16.6% increases in mAP0.5:0.95 and mAR0.5:0.95 metrics, respectively. These experimental results confirm the enhanced detection capability of our module for agricultural pest identification tasks.

Following the methodological recommendations from the GCT attention mechanism’s authors, this study systematically evaluates the impact of different module integration strategies and hybrid attention configurations, as detailed in Table 7. All experimental comparisons employ the SGD optimizer with an enhanced Warmup CosineAnnealing learning rate scheduler, combined with various attention mechanism (AM) implementations. The Layer23 configuration integrates GCT modules before the final two convolutional layers in each ResBlock, while Layer123 extends this integration across all convolutional operations within the ResBlock architecture. Experimental results demonstrate that the Layer123 implementation achieves optimal performance, validating our module’s effectiveness when comprehensively applied throughout the network structure. The proposed model achieves an mAP0.5:0.95 of 0.819, outperforming both GCT + SAM and Layer23 configurations by 0.4% and 0.7%, respectively, while showing a 1.2 percentage point improvement over GCT + SAM. Analysis of mAP0.5, mAP0.75, and mAP0.5:0.95 metrics confirms enhanced detection accuracy and recall, particularly under stringent IoU threshold conditions, demonstrating our method’s superior performance in precise localization tasks. The experimental results indicate that introducing EIoU can enhance the model’s ability to extract fine-grained discriminative features.

Table 8 shows a comparison of the detection effects of different models in each category. Table analysis reveals that the proposed GC-Faster RCNN model demonstrates improved detection accuracy across 19 categories, despite minor performance declines in six specific categories (20096, 20158, 20215, 20262, 20265, and 20338). For the highly similar categories 20143 and 20266, GC-Faster RCNN achieves accuracy rates of 0.823 and 0.893, representing improvements of 2.1% and 1.3% over the best-performing C-Faster RCNN and G-Faster RCNN models, respectively. These results demonstrate GC-Faster RCNN’s enhanced capability in distinguishing between visually similar categories, highlighting its superior feature discrimination performance. The sizes of 20015, 20072, and 30067 category objects are smaller than that of other categories, and our method achieved mAP0.5:0.95 of 0.837, 0.891, and 0.720 for these three categories, respectively, which were 0.5%, 1.1%, and 0.4% higher than the second-best-performing G-Faster RCNN. These results demonstrate GC-Faster RCNN’s enhanced capability in handling multi-scale detection challenges, effectively addressing variations in object sizes across different categories.

### 3.4. Visual Experiment

**Figure 10:**
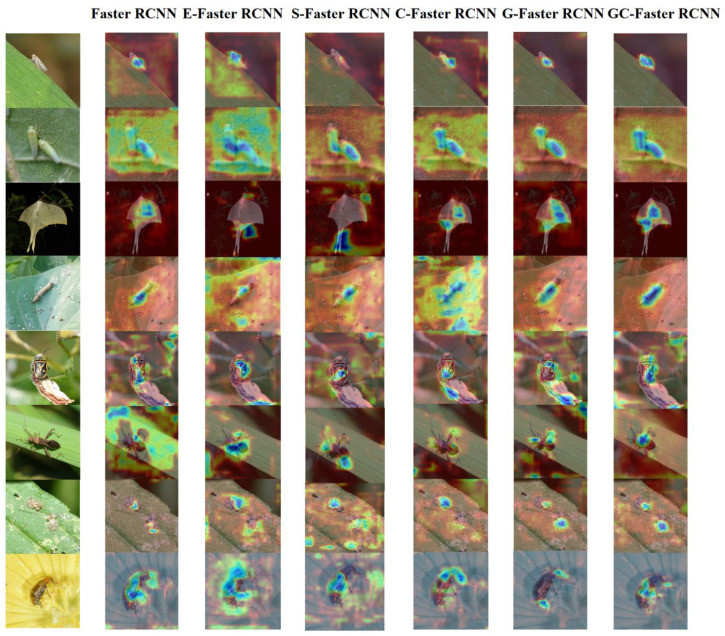
Visual feature map for Gard-CAM.

Using Grad-CAM visualization techniques, we generated comparative feature maps to analyze the models’ attention patterns. Figure 10 presents a comprehensive collection of these visual feature maps, highlighting the distinctive attention distributions across different categories and model architectures. The visualization heatmaps reveal significant background noise interference in conventional models, adversely affecting object recognition accuracy. Notably, GC-Faster RCNN demonstrates superior performance by precisely localizing target objects while effectively suppressing irrelevant background features. Furthermore, in multi-object scenarios, the model exhibits enhanced capability to simultaneously focus on discriminative features across multiple targets, demonstrating robust multi-object recognition performance. The Grad-CAM heatmap confirms the effectiveness of our method once again.

**Figure 11:**
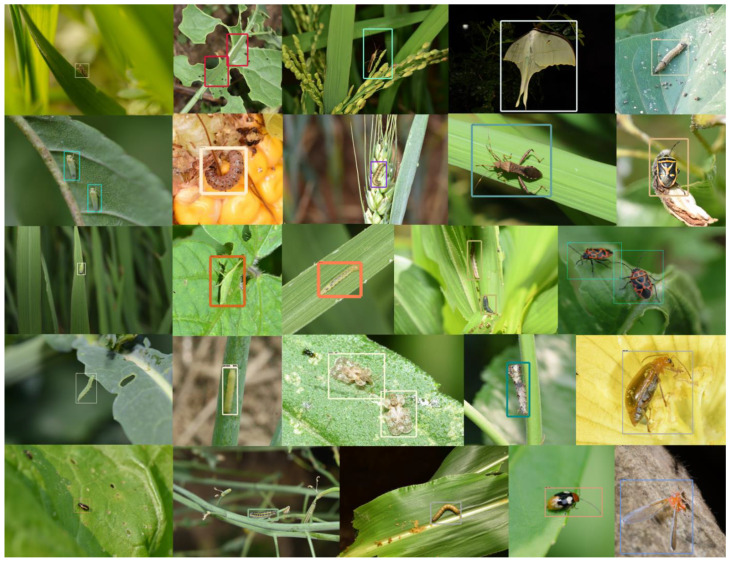
The visualization of detection results of GC-Faster RCNN.

Figure 11 shows the detection results of GC-Faster RCNN for each category. The dataset exhibits significant intra-category size variations and high inter-category visual similarities among objects. Visualization results demonstrate that our proposed agricultural pest detection model effectively addresses these challenges, achieving robust detection performance across varying object scales and maintaining high discriminative capability for visually similar categories.

## 4. Discussion

To address the challenges of agricultural pest detection in complex field environments, this study proposes GC-Faster RCNN—a hybrid attention-enhanced framework that integrates GCT and CBAM to improve multi-scale feature representation for robust pest recognition. By embedding the GCT-CBAM hybrid attention mechanism into the ResNet-50 backbone with FPN integration, the proposed method significantly enhances detection performance for multi-scale objects and visually similar categories, achieving superior recognition accuracy. During model training, the integration of a cosine annealing learning rate scheduler with warmup initialization and the EIoU loss function significantly accelerates convergence while enhancing training stability. Comparative experiments under identical parameters and experimental conditions demonstrate the superior performance of the GC-Faster RCNN, achieving mAP0.5:0.95 improvements of 16.7%, 4.8%, 3.8%, 1.6%, and 0.5% over Faster RCNN, ECA, SE, CBAM, and GCT-based models, respectively, with significant accuracy enhancement.

The proposed GC-Faster RCNN model achieves accurate detection of 25 agricultural pest species, including leafhoppers, in field environments, demonstrating significant potential for advancing agricultural pest management through information technology. However, the Insect25 dataset primarily consists of clear, sparse images, which do not fully represent the challenging scenarios of dense and occluded pest populations commonly encountered in real-world conditions. In future research, regarding dataset construction, we will incorporate image modalities related to occluded and densely distributed pests, with a focus on enhancing the capability of the proposed model to handle occluded, densely clustered, and partially obscured pests, thus improving its accuracy and robustness in complex agricultural scenarios. Furthermore, efforts will focus on developing fast-inference models and reducing parameter counts through model compression techniques, enabling practical deployment in agricultural field settings to significantly enhance production efficiency. These advancements aim to establish a reliable foundation for intelligent plant protection systems, offering valuable insights for practical agricultural applications.

## Figures and Tables

**Table 1:** Comparison of different datasets.

Dataset	Task	Scale	Resolution	Categories	Number	Source
IP102 [21]	Detection	Large-scale	Low	102	75,222	Internet
Tomato pests [22]	Classification	Multi-scale	Low	8	609	Internet
Orchard pests [23]	Classification	Multi-scale	Low	6	1568	Internet
Insect25	Detection	Multi-scale	High	25	18,349	Real scenes

**Table 2:** The Insect25 dataset.

Class	Name	Number	Class	Name	Number
20015	*Psammotettix striatus* (Linnaeus)	529	20262	*Corythucha marmorata* (Uhler)	944
20017	*Singapora shinshana* (Matsumura)	555	20265	*Pieris rapae* (Linnaeus)	488
20049	*Leptocorisa acuta* (Thunberg)	264	20266	*Eurydema dominulus* (Scopoli)	696
20062	*Actias ningpoana* Felder	281	20302	*Bombyx mandarina* (Moore)	234
20072	*Plutella xylostella* (Linnaeus)	450	20332	*Phyllotreta striolata* (Fabricius)	2210
20096	*Spodoptera litura* (Fabricius)	1234	20334	*Aulacophora indica* (Gmelin)	635
20127	*Conogethes punctiferalis* (Guenée)	340	20338	*Mythimna separata* (Walker)	1871
20137	*Helicoverpa armigera* (Hübner)	726	20341	*Nephotettix cincticeps* (Fabricius)	773
20143	*Eurydema gebleri* Kolenati	478	30023	*Spodoptera frugiperda* (J. E. Smith)	1147
20158	*Riptortus pedestris* (Fabricius)	969	30067	*Monolepta signata* (Olivier)	1205
20188	*Spodoptera exigua* (Hübner)	257	30106	*Pontia edusa* (Fabricius)	346
20202	*Atractomorpha sinensis* Bolvar	920	20262	*Corythucha marmorata* (Uhler)	944
20215	*Cnaphalocrocis medinalis* (Guenée)	278	20265	*Pieris rapae* (Linnaeus)	488
20231	*Diostrombus politus* Uhler	519	20266	*Eurydema dominulus* (Scopoli)	696
			20302	*Bombyx mandarina* (Moore)	234

**Table 3:** Performance comparison of different models.

Model	mAP_0.5_	mAP_0.75_	mAP_0.5:0.95_	mAR_0.5:0.95_
Faster RCNN [10]	0.925	0.735	0.611	0.674
YOLOv7 [39]	0.924	0.912	0.700	0.835
E-Faster RCNN	0.981	0.924	0.756	0.791
S-Faster RCNN	0.974	0.918	0.757	0.794
C-Faster RCNN	0.977	0.921	0.761	0.795
G-Faster RCNN	0.978	0.917	0.769	0.796
GC-Faster RCNN (ours)	0.970	0.939	0.819	0.840

**Table 4:** Performance comparison of different attention mechanisms.

AM	mAP_0.5_	mAP_0.75_	mAP_0.5:0.95_	mAR_0.5:0.95_
Original	0.925	0.735	0.611	0.674
ECA	0.981	0.924	0.756	0.791
SE	0.974	0.918	0.757	0.794
CBAM	0.977	0.921	0.761	0.795
GCT	0.978	0.917	0.769	0.796
Ours	0.963	0.923	0.779	0.823

**Table 5:** Comparison of optimization functions with different learning rates.

AM	Optimizer	IoU	mAP_0.5_	mAP_0.75_	mAP_0.5:0.95_	mAR_0.5:0.95_
Original	SGD + MultiStep + Warmup	-	0.931	0.749	0.655	0.694
	SGD + CosineAnnealing + Warmup		0.928	0.755	0.658	0.688
ECA	SGD + MultiStep + Warmup	-	0.980	0.936	0.762	0.804
	SGD + CosineAnnealing + Warmup		0.971	0.935	0.769	0.810
SE	SGD + MultiStep + Warmup	-	0.988	0.921	0.775	0.796
	SGD + CosineAnnealing + Warmup		0.988	0.922	0.776	0.804
CBAM	SGD + MultiStep + Warmup	-	0.978	0.930	0.786	0.796
	SGD + CosineAnnealing + Warmup		0.978	0.933	0.794	0.812
GCT	SGD + MultiStep + Warmup	-	0.972	0.938	0.807	0.837
	SGD + CosineAnnealing + Warmup		0.972	0.943	0.810	0.834
Ours	SGD + MultiStep + Warmup	-	0.978	0.942	0.814	0.837
	SGD + CosineAnnealing + Warmup		0.972	0.936	0.817	0.841

**Table 6:** Comparison of EIoU added to different models.

AM	Optimizer	IoU	mAP_0.5_	mAP_0.75_	mAP_0.5:0.95_	mAR_0.5:0.95_
Original	SGD + CosineAnnealing + Warmup	- EIoU	0.928 0.928	0.755 0.753	0.658 0.652	0.688 0.703
ECA	SGD + CosineAnnealing + Warmup	- EIoU	0.971 0.971	0.935 0.936	0.769 0.771	0.810 0.811
SE	SGD + CosineAnnealing + Warmup	- EIoU	0.988 0.987	0.922 0.926	0.776 0.781	0.804 0.808
CBAM	SGD + CosineAnnealing + Warmup	- EIoU	0.978 0.975	0.933 0.934	0.794 0.803	0.812 0.834
GCT	SGD + CosineAnnealing + Warmup	- EIoU	0.972 0.970	0.943 0.938	0.810 0.814	0.834 0.835
Ours	SGD + CosineAnnealing + Warmup	- EIoU	0.972 0.970	0.936 0.939	0.817 0.819	0.841 0.840

**Table 7:** Performance of different improved models.

Method	AM	IoU	mAP_0.5_	mAP_0.75_	mAP_0.5:0.95_	mAR_0.5:0.95_
Layer23	GCT + SAM	EIoU	0.971	0.939	0.807	0.830
Layer23	Ours	EIoU	0.971	0.941	0.812	0.840
Layer123	GCT + SAM	EIoU	0.972	0.936	0.815	0.841
Layer123	Ours	EIoU	0.970	0.939	0.819	0.840

**Table 8:** Test results of different models in each category.

Class	Model	mAP _0.5:0.95_	mAR _0.5:0.95_	Class	Model	mAP _0.5:0.95_	mAR _0.5:0.95_
20015	Faster RCNN	0.677	0.738	20231	Faster RCNN	0.759	0.796
	E-Faster RCNN	0.657	0.740		E-Faster RCNN	0.841	0.866
	S-Faster RCNN	0.754	0.823		S-Faster RCNN	0.855	0.878
	C-Faster RCNN	0.848	0.887		C-Faster RCNN	0.898	0.924
	G-Faster RCNN	0.830	0.873		G-Faster RCNN	0.895	0.922
	GC-Faster RCNN	0.837	0.871		GC-Faster RCNN	0.911	0.935
20017	Faster RCNN	0.622	0.683	20262	Faster RCNN	0.697	0.550
	E-Faster RCNN	0.654	0.706		E-Faster RCNN	0.702	0.555
	S-Faster RCNN	0.728	0.782		S-Faster RCNN	0.669	0.543
	C-Faster RCNN	0.786	0.832		C-Faster RCNN	0.725	0.589
	G-Faster RCNN	0.791	0.834		G-Faster RCNN	0.755	0.573
	GC-Faster RCNN	0.798	0.834		GC-Faster RCNN	0.747	0.573
20049	Faster RCNN	0.516	0.573	20265	Faster RCNN	0.666	0.669
	E-Faster RCNN	0.550	0.615		E-Faster RCNN	0.760	0.730
	S-Faster RCNN	0.628	0.699		S-Faster RCNN	0.821	0.775
	C-Faster RCNN	0.732	0.775		C-Faster RCNN	0.847	0.793
	G-Faster RCNN	0.715	0.767		G-Faster RCNN	0.879	0.812
	GC-Faster RCNN	0.726	0.767		GC-Faster RCNN	0.878	0.814
20062	Faster RCNN	0.761	0.804	20266	Faster RCNN	0.763	0.807
	E-Faster RCNN	0.810	0.849		E-Faster RCNN	0.829	0.854
	S-Faster RCNN	0.827	0.873		S-Faster RCNN	0.852	0.875
	C-Faster RCNN	0.887	0.913		C-Faster RCNN	0.877	0.902
	G-Faster RCNN	0.906	0.928		G-Faster RCNN	0.880	0.902
	GC-Faster RCNN	0.925	0.941		GC-Faster RCNN	0.893	0.922
20072	Faster RCNN	0.701	0.745	20302	Faster RCNN	0.733	0.778
	E-Faster RCNN	0.794	0.831		E-Faster RCNN	0.741	0.786
	S-Faster RCNN	0.829	0.865		S-Faster RCNN	0.786	0.821
	C-Faster RCNN	0.881	0.898		C-Faster RCNN	0.810	0.850
	G-Faster RCNN	0.880	0.909		G-Faster RCNN	0.830	0.874
	GC-Faster RCNN	0.891	0.913		GC-Faster RCNN	0.837	0.876
20096	Faster RCNN	0.650	0.749	20332	Faster RCNN	0.677	0.694
	E-Faster RCNN	0.781	0.811		E-Faster RCNN	0.649	0.690
	S-Faster RCNN	0.819	0.864		S-Faster RCNN	0.763	0.768
	C-Faster RCNN	0.861	0.906		C-Faster RCNN	0.795	0.798
	G-Faster RCNN	0.868	0.903		G-Faster RCNN	0.793	0.792
	GC-Faster RCNN	0.867	0.900		GC-Faster RCNN	0.808	0.811
20127	Faster RCNN	0.634	0.700	20334	Faster RCNN	0.598	0.634
	E-Faster RCNN	0.635	0.669		E-Faster RCNN	0.578	0.610
	S-Faster RCNN	0.717	0.769		S-Faster RCNN	0.687	0.712
	C-Faster RCNN	0.679	0.774		C-Faster RCNN	0.651	0.720
	G-Faster RCNN	0.744	0.800		G-Faster RCNN	0.663	0.717
	GC-Faster RCNN	0.754	0.811		GC-Faster RCNN	0.663	0.727
20137	Faster RCNN	0.572	0.664	20338	Faster RCNN	0.537	0.702
	E-Faster RCNN	0.624	0.700		E-Faster RCNN	0.599	0.732
	S-Faster RCNN	0.711	0.782		S-Faster RCNN	0.715	0.776
	C-Faster RCNN	0.808	0.850		C-Faster RCNN	0.758	0.819
	G-Faster RCNN	0.789	0.842		G-Faster RCNN	0.776	0.819
	GC-Faster RCNN	0.833	0.890		GC-Faster RCNN	0.753	0.792
20143	Faster RCNN	0.684	0.709	20341	Faster RCNN	0.730	0.778
	E-Faster RCNN	0.692	0.747		E-Faster RCNN	0.707	0.770
	S-Faster RCNN	0.790	0.816		S-Faster RCNN	0.799	0.834
	C-Faster RCNN	0.802	0.845		C-Faster RCNN	0.867	0.900
	G-Faster RCNN	0.799	0.831		G-Faster RCNN	0.847	0.882
	GC-Faster RCNN	0.823	0.849		GC-Faster RCNN	0.853	0.887
20158	Faster RCNN	0.685	0.726	30023	Faster RCNN	0.670	0.759
	E-Faster RCNN	0.778	0.817		E-Faster RCNN	0.760	0.816
	S-Faster RCNN	0.799	0.836		S-Faster RCNN	0.799	0.845
	C-Faster RCNN	0.860	0.891		C-Faster RCNN	0.816	0.850
	G-Faster RCNN	0.872	0.904		G-Faster RCNN	0.844	0.883
	GC-Faster RCNN	0.870	0.902		GC-Faster RCNN	0.847	0.847
20188	Faster RCNN	0.606	0.695	30067	Faster RCNN	0.625	0.665
	E-Faster RCNN	0.689	0.745		E-Faster RCNN	0.595	0.633
	S-Faster RCNN	0.748	0.814		S-Faster RCNN	0.698	0.713
	C-Faster RCNN	0.831	0.865		C-Faster RCNN	0.681	0.724
	G-Faster RCNN	0.812	0.852		G-Faster RCNN	0.716	0.750
	GC-Faster RCNN	0.830	0.868		GC-Faster RCNN	0.720	0.726
20202	Faster RCNN	0.533	0.606	30106	Faster RCNN	0.713	0.739
	E-Faster RCNN	0.609	0.661		E-Faster RCNN	0.828	0.833
	S-Faster RCNN	0.685	0.725		S-Faster RCNN	0.818	0.825
	C-Faster RCNN	0.727	0.779		C-Faster RCNN	0.869	0.868
	G-Faster RCNN	0.732	0.785		G-Faster RCNN	0.864	0.870
	GC-Faster RCNN	0.733	0.763		GC-Faster RCNN	0.874	0.876
20215	Faster RCNN	0.670	0.726				
	E-Faster RCNN	0.746	0.786				
	S-Faster RCNN	0.804	0.834				
	C-Faster RCNN	0.837	0.880				
	G-Faster RCNN	0.857	0.890				
	GC-Faster RCNN	0.848	0.885				

## Data Availability

Data are contained within the article.

## Abbreviations

GC	Gated and CBAM
RCNN	Region-based Convolutional Neural Networks
